# Cryo-EM structure of the nucleocapsid-like assembly of respiratory syncytial virus

**DOI:** 10.1038/s41392-023-01602-5

**Published:** 2023-08-22

**Authors:** Yan Wang, Chong Zhang, Yongbo Luo, Xiaobin Ling, Bingnan Luo, Guowen Jia, Dan Su, Haohao Dong, Zhaoming Su

**Affiliations:** The State Key Laboratory of Biotherapy, Frontiers Medical Center of Tianfu Jincheng Laboratory, Department of Geriatrics and National Clinical Research Center for Geriatrics, West China Hospital, Sichuan University, Chengdu, Sichuan 610044 China

**Keywords:** Structural biology, Biophysics

## Abstract

Respiratory syncytial virus (RSV) is a nonsegmented, negative strand RNA virus that has caused severe lower respiratory tract infections of high mortality rates in infants and the elderly, yet no effective vaccine or antiviral therapy is available. The RSV genome encodes the nucleoprotein (N) that forms helical assembly to encapsulate and protect the RNA genome from degradation, and to serve as a template for transcription and replication. Previous crystal structure revealed a decameric ring architecture of N in complex with the cellular RNA (N-RNA) of 70 nucleotides (70-nt), whereas cryo-ET reconstruction revealed a low-resolution left-handed filament, in which the crystal monomer structure was docked with the helical symmetry applied to simulate a nucleocapsid-like assembly of RSV. However, the molecular details of RSV nucleocapsid assembly remain unknown, which continue to limit our complete understanding of the critical interactions involved in the nucleocapsid and antiviral development that may target this essential process during the viral life cycle. Here we resolve the near-atomic cryo-EM structure of RSV N-RNA that represents roughly one turn of the helical assembly that unveils critical interaction interfaces of RSV nucleocapsid and may facilitate development of RSV antiviral therapy.

## Introduction

Human respiratory syncytial virus (RSV) is a nonsegmented, negative strand RNA virus (NSV) that causes lethal lower respiratory tract infections and continues to pose health threats to infants, the elderly, and people with compromised immune systems worldwide.^1–4^ It is estimated that almost all children have been infected by RSV by the age of two, leading to an annual estimation of 3.6 million hospitalizations and more than 100,000 deaths globally for children under 5-year-old.^3^ Aside from high mortality rate in newborn infants, severe respiratory diseases caused by RSV are also associated with the development of memory-related brain networks, which could impair early language learning ability in infants and young children, leading to long-term language learning difficulties.^5^ No vaccine or antiviral therapy is currently available to combat RSV infections.^6,7^

**Fig. 1:**
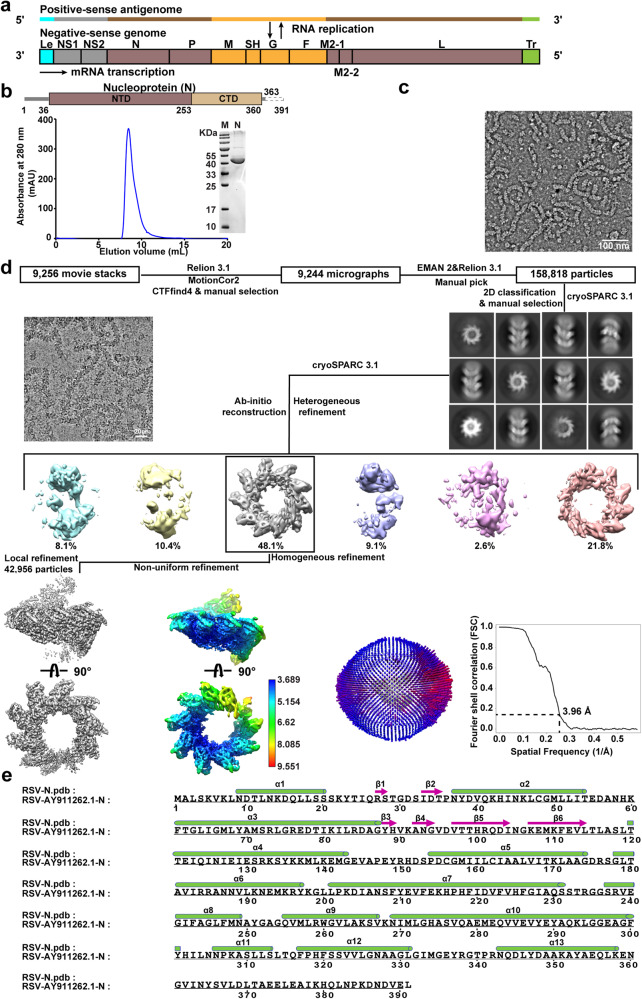
Schematic view of the RSV genome, cryo-EM data processing workflow for N-RNA complexes and N protomer secondary structure. **a** The positive-sense antigenome and its negative-sense genome. **b** Schematic view of N: the full-length N contains 391 amino acids, and consists of NTD and CTD at the core, with extensions “N-terminal” and “C-terminal” on both sides. Gel filtration chromatography (Superdex^TM^ 200 Increase 10/300 GL column, Cytiva) elution profile of N protein in a buffer containing 150 mM NaCl. Inset: Coomassie-stained SDS-PAGE of corresponding peak fractions from gel filtration. **c** Representative negative stain image. Scale bar, 100 nm. **d** Representative cryo-EM micrograph, 2D class averages, 3D classification and 3D refinement, local resolution map and angular distribution. The nominal resolution of RSV N-RNA complex is estimated by the 0.143 criterion of the Fourier shell correlation (FSC) curve. Scale bar, 20 nm. **e** The N protomer consists of 13 α-helices and 6 β-strands. The green cylinder indicates α-helix, whereas the plum-red arrow indicates β-strand

RSV belongs to *Pneumoviridae* in the *Mononegavirales* order, and the genome is about 15.2 kilobases that contains 10 genes encoding 11 proteins: non-structural proteins 1 and 2 (NS1 and NS2) that together regulate evasion from the host immune systems;^8,9^ nucleoprotein (N) that assembles into nucleocapsid that wraps around and protects the viral genome; phosphoprotein (P) that normally exists as a homo tetramer that could serve as the chaperone of N to regulate N-RNA binding and to deliver N and the RNA polymerase to the RNA genome;^10^ large RNA polymerase (L) that forms the RNA-dependent RNA polymerase (RdRp) complex together with N and P to perform transcription and replication; external transmembrane attachment glycoproteins (G) and fusion protein (F) that are responsible for virus entry by recognizing cell surface receptors and facilitating membrane fusions; small hydrophobic protein (SH) that is an ion channel proposed to involve in the regulation of host cell apoptosis;^11^ and matrix proteins M that forms a matrix layer to support the viral envelope, M2-1 that associates M with the nucleocapsid,^12^ and M2-2 that involves in transcription/replication regulations (Fig. 1a).^13,14^ The N protein is highly conserved, with up to 96% homology at the amino acid level across different strains.^15^ N consists of 391 amino acids with N-terminal domain (NTD) (residues 36–253) and C-terminal domain (CTD) (residues 254–360), which is one of the most abundant proteins that forms the helical assembly with viral RNAs to provide protection from degradation and to participate in transcription and replication as the template.^16^ The selective binding of N to viral RNAs is facilitated by P that binds to and prevent N from binding to non-viral genomic RNA or self-aggregation, which is especially important for the packaging of viral particles.^10,17^ In the late stage of RSV infection, the N-terminal 110 amino acids of the M protein can interact with N in the presence of M2-1 to inhibit viral transcription, thereby initiating the assembly and budding process.^18^ In addition, N protein has many important functions, including participation in the interaction between virus and host proteins, interference with the formation of immune synapses, evasion of the innate host immune system, and regulation of transcription/replication as part of the RdRp complex.^19^ These highly conserved and important biological functions have made N protein as an attractive target, and in-depth structural and functional studies of N will provide insights that may facilitate development of novel anti-RSV therapy.

The helical and circular nucleocapsid-like assembly of RSV have been previously observed by negative staining, and projection averages indicated that the ring-like structure contains 10 or 11 RSV N protomers.^20^ Previous crystal structure of the RSV N protein in complex with the cellular RNA (N-RNA) revealed crystal packing of a decameric ring architecture, with the 7-nucleotide (7-nt) RNA bound to the positively charged groove between the NTD and CTD of N.^21^ Subsequent cryo-electron tomography (cryo-ET) studies both in vitro and in virions revealed a low-resolution left-handed filamentous assembly,^22–24^ in which the crystal structure was docked with the helical symmetry applied to simulate the nucleocapsid-like assembly.^22^ However, the lack of high-resolution structure of RSV nucleocapsid assembly continues to limit our understanding of the essential interaction interfaces in the nucleocapsid and poses challenges on antiviral development targeting this RSV N-RNA complex.

In this study, we determine the structure of roughly one turn of RSV nucleocapsid assembly by single particle cryo-electron microscopy (cryo-EM) at 3.96 Å resolution. Residues Y88 and D221 of N form hydrogen bond and salt bridge with R234 of the adjacent N + 1, whereas Q26 of N also forms hydrogen bond with Y38 of N + 1; hydrophobic interactions of the α-helix 1 (α1) and loop 1 (L1) of N with multiple α-helices of N + 1 together comprise the protein interaction interfaces. Each N binds to 7-nt RNA in positively charged groove with interactions between residues K170, R184, R185, S313, T315, Y337 and the phosphate backbone. Additionally, we compare with previously reported interaction interfaces and identify novel protein-protein and protein-RNA interactions. Together our results elucidate the interaction interfaces crucial for RSV nucleocapsid assembly that may enable development of potential RSV antiviral therapy.

## Results

### Cryo-EM structure of the RSV nucleocapsid assembly

**Fig. 2:**
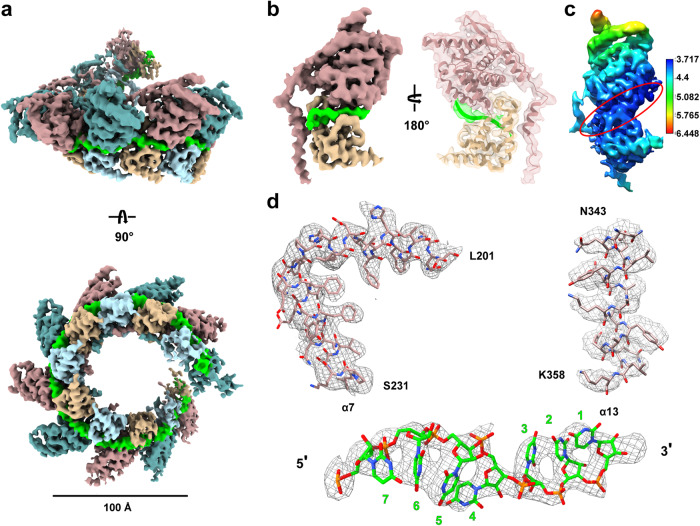
Cryo-EM structures and models of RSV nucleocapsid-like assembly. **a** Side and top views of the cryo-EM reconstruction of ~1 turn of RSV nucleocapsid-like assembly, with the RNA highlighted in green, and N protomers highlighted in blue and brown, respectively. Scale bar, 100 Å. **b** Cryo-EM map and model of one N protomer. **c** Local resolution map of N protomer. Red ellipse indicates RNA density with local resolution at better than 4 Å. **d** Representative density maps and models of α7, α13 and RNA nucleotides reveal resolved side chain and nucleotide density

We prepared the RSV nucleocapsid assembly by recombinant expression of the full-length N in HEK293 cells (Fig. 1b). Purification under 150 mM NaCl condition resulted in the peak elution volume of 8.47 mL in gel filtration chromatography that typically indicates the presence of assembly or oligomers (Fig. 1b). The presence of cellular RNA in the assembly was confirmed by measuring the absorbance of 260/280 as 1.13. Negative stain images revealed relatively short and curved filaments, suggesting high flexibility of the RSV nucleocapsid assembly (Fig. 1c). Nevertheless, we collected cryo-EM data and the two-dimensional (2D) classification revealed better features in the center of the segmented filaments. Final three-dimensional (3D) reconstruction yielded the RSV nucleocapsid of roughly one left-handed helical turn of 10 N protomers at 3.96 Å resolution, with the top view resembling the decameric ring architecture as reported in previous crystal structure (Fig. 1d). Modeling of individual N resembled the previous crystal structure with well resolved density for protein side chains and RNA bases and similar secondary structure (Figs. 1e and 2). All nucleotides were modeled as uridine due to the random composition of cellular RNA, with each N bound to 7-nt RNA.

### Protein-protein interaction surfaces in the RSV nucleocapsid assembly

**Fig. 3:**
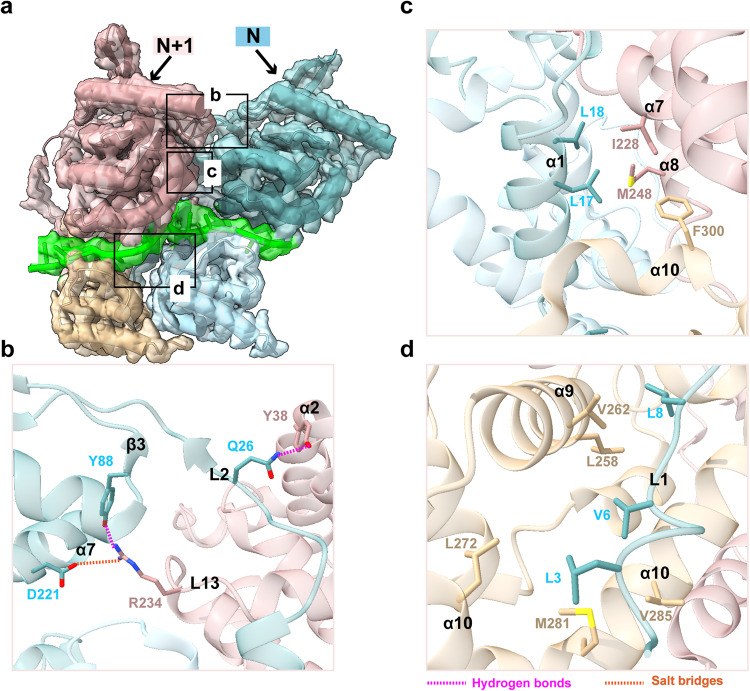
Protein-protein interactions in RSV N-RNA complex. **a** Two adjacent N protomer models in light blue and rosy brown fitted into the N-RNA density map. Black boxes outline zoom-in regions. **b** Interactions between Y88 and D221 of N and R234 of N + 1, and interactions between Q26 of N and Y38 of N + 1. **c** Hydrophobic interactions between α1 of N and multiple residues of N + 1. **d** Hydrophobic interactions between L1 of N and multiple residues of N + 1. Magenta dashed lines indicate hydrogen bonds and dark orange dashed lines indicate salt bridge

Given the less compact assembly form, we reexamined the protein-protein and protein-RNA interaction interfaces (Fig. 3a). Previous study indicated R234 as a key residue for nucleocapsid assembly and virus replication, which was hampered upon introduction of R234A point mutation.^22^ We observed that Y88 in β3 and D221 in α7 of N interact with R234 in L13 of N + 1 via hydrogen bond and salt bridge, validating the key role of R234 in interaction interface (Fig. 3b). In addition, we identified interactions between Q26 in L2 of N and Y38 in α2 of N + 1 through hydrogen bond as predicted by previous study (Fig. 3b).^25^ Y38 phosphorylation has been previously demonstrated to modulate RSV transcription and replication by reducing the nucleocapsid template activity, while making the nucleocapsid more compact to potentially promote packaging, as Y38 phosphorylation has also been detected in RSV virions.^25^ The absence of Y38 phosphorylation in our structure suggests that viral transcription and replication likely utilize less compact nucleocapsid template. Finally, we observed hydrophobic interactions between α1 and L1 of N and multiple hydrophobic regions of N + 1, further enhancing the protein-protein interfaces (Fig. 3c, d).

### Protein-RNA interaction interfaces

**Fig. 4:**
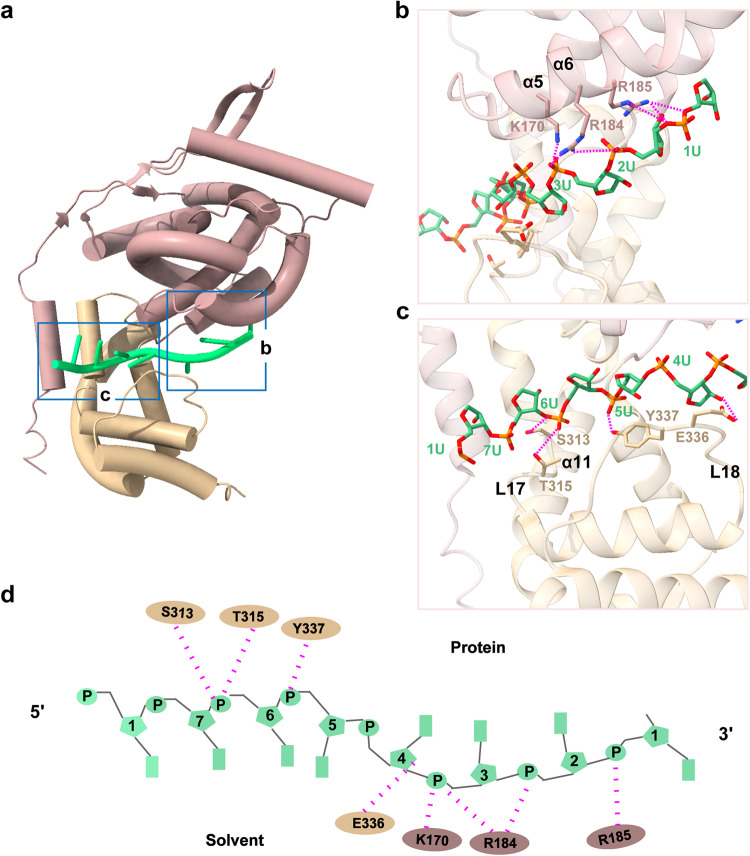
Protein-RNA interactions in RSV N-RNA complex. **a** One N protomer model fitted into the N-RNA density map. Blue boxes outline zoom-in regions. **b**, **c** One N protomer bound to RNA. The interactions of NTD (**b**) and CTD (**c**) with RNA are shown, respectively. **d** Schematic diagram of protein-RNA interface. RNA nucleotides are shown in green, residues from the N- and C-terminal lobes are shown in rosy brown and tan

**Fig. 5:**
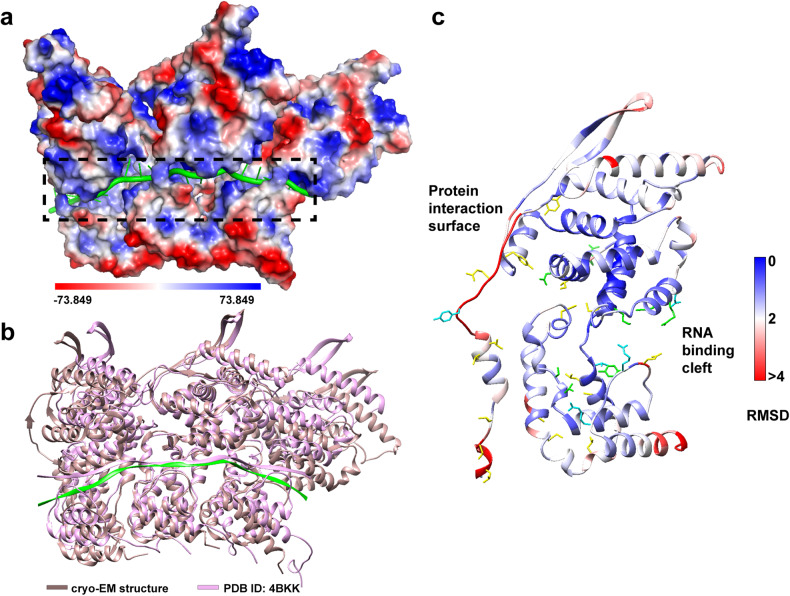
Electropotential distribution and comparison with reported structures of cryo-EM structure of RSV N-RNA complex. **a** Electropotential map of three N protomers bound to RNA. RNA is shown in green. The electrostatic potential distribution was generated using PyMOL. **b** Comparison of three N protomers between our cryo-EM structure and the simulated NC-like structure with crystal structures fitting into the low-resolution cryo-ET reconstruction. Superposition of three N protomers from the cryo-EM structure (RNA highlighted in green and N protomers highlighted in rosy brown) and simulated structure (pink, PDB 4BKK) aligned to the central N protomer. **c** Comparison of RMSD per residue and interaction residues between the cryo-EM and crystal structure (PDB 2WJ8). The model is colored based on RMSD per residue, interaction residues found only in the cryo-EM structure are shown in yellow, residues found only in the reported structures are shown in cyan, and residues found in both structures are shown in green

The RSV-N protein consists of the N-terminal lobe and the C-terminal lobe linked by L14 that is conserved among all negative strand RNA viruses.^26^ The viral RNA genome is encapsulated in the cleft formed between the two lobes, with stacked bases resembling one strand of the A-form RNA double-helix except that some bases point inward while the rest point outward.^27^ In our nucleocapsid-like assembly structure, we observed seven nucleotides encapsulated in the overall positively charged cleft of each N protomer, with four bases facing outward and three facing inward (Fig. 4a–c). The positively charged residues K170, R184 and R185 in N-terminal lobe form electrostatic interactions with the phosphate backbone of the nucleotides pointing inward, whereas S313, T315 and Y337 in C-terminal lobe form hydrogen bonds with the phosphate backbone of the nucleotides pointing outward to the solvent (Figs. 4d, 5a). The key residues identified in protein-RNA interaction interfaces in our cryo-EM structure are fewer than those in the previous ring-like crystal structure, probably because the RSV nucleocapsid-like assembly is less compact.

### Comparison with the reported structure of RSV N protein

Three adjacent N protomers showed an overall good agreement of the central N with r.m.s. deviation (RMSD) of 2.04 Å (Fig. 5b), whereas local regions could deviate as much as 7 Å (Fig. 5c). Larger translations were observed on both the N-1 and N + 1 with RMSD of 7.63 Å and 8.93 Å compared to the simulated structure of three N protomer crystal structures fitted into the low-resolution cryo-ET reconstruction (Fig. 5b). Comparison between cryo-EM and crystal structures revealed that more protein-protein interaction residues were identified in the cryo-EM model. Additional protein-RNA interaction residues observed in the crystal structure may be caused by the loosely compact nucleocapsid-like assembly in cryo-EM structure (Fig. 5c).

## Discussion

Nucleoproteins of all nonsegmented NSV encapsulate the viral RNA genomes and form helical nucleocapsid assembly in order to protect viral RNAs of different lengths from degradation and to serve as templates for transcription and replication. Albeit RSV is one of the most important pathogens infecting infants with no currently available treatment, recently developed RNAi therapy has shown decent effect in a mouse model infected with RSV.^28^ A small interfering RNA (siRNA, ALN-RSV01) with strong antiviral effects on both RSV type A and B viruses was designed by targeting the mRNA in N protein that is highly conserved in known RSV clades.^15,29^ Development of antiviral small molecules such as RSV604 by Chapman et al. has demonstrated that structure-based drug design targeting the N protein to inhibit the formation of nucleocapsid could be a new strategy for the development of antiviral compounds.^30,31^ However, these applications associated with RSV N protein remain to be limited partially due to the dearth of high-resolution structure of RSV nucleocapsid assembly. Prior to this study, the only available structural information was the high-resolution crystal structure of the ring-like N-RNA complex and the low-resolution cryo-ET reconstruction of the left-handed helical assembly,^21,22^ in which the N protomer being docked into the low-resolution helical assembly may not be the most functionally relevant form.^32^

During NSV viral RNA synthesis, the newly formed viral RNA is encapsulated by RNA-free monomeric N protein, either with or without the help from other viral chaperone proteins such as phosphoprotein, and assemble into nucleocapsid to serve as the template for replication.^33^ During this process, the nucleocapsid has been suggested to be dynamic in order to allow RNA template readthrough by the transcription or replication complexes.^34^ It was previously found that flexible and less compact nucleocapsid assembly enables interactions of the template RNA sequence with the replication complex during transcription and replication.^26^ Our cryo-EM structure reveals the loosely compact RSV nucleocapsid-like assembly that could potentially serve as the replication template. Multiple key residues and interfaces of protein-protein and protein-RNA interactions that are essential to forming the assembly of RSV nucleocapsid are identified, some of which were also functionally validated by previous mutation experiments (Figs. 3, 4).^21,22,25^ For example, Y337 variant disrupted the formation of nucleocapsid;^21,35^ Y38 phosphorylation can enhance the interaction between N protomers in nucleocapsid assembly, whereas dephosphorylation is required to optimize transcription or replication because Y38D decreased the template activity;^25^ R234A affects viral RNA synthesis.^22^

**Fig. 6:**
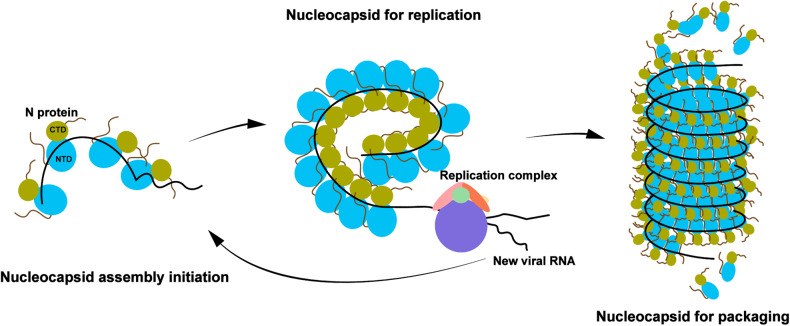
Cartoon of the RSV nucleocapsid assembly. N proteins encapsulate viral RNA to form either loose helical assembly as the template for replication and transcription, or more compact assembly for virion packaging. Created with Adobe Illustrator

Depending on the size of the RNA binding cleft, the number of nucleotides bound to each N asymmetric unit in the phylum *Negarnaviricota* ranges from 3 to 11,^27^ for example the Hantaan Virus N binds 3 nucleotides,^36^ the measles virus N binds 6,^37^ the human metapneumovirus N binds 7,^38^ the vesicular stomatitis virus N binds 9,^39^ the Bunyamwera virus N binds 10^40^ and La crosse orthobunyavirus N binds 11.^41^ Further analysis of the interaction between the N protein and RNA in the RSV N structure revealed that positions 3 and 6 interact more with the N protein than the rest of the positions, and the total number of interaction sites was less than that found in the crystal structure likely due to the extended, loosely compact form of this assembly (Figs. 4d, 5c). Seven nucleotides are bound to the positively charged cleft with three bases pointing inward stabilized by electrostatic interactions from positively charged residues in the N-terminal lobe, and four bases pointing outward stabilized by hydrogen bonds from neutral residues in the C-terminal lobe (Figs. 4, 5). Other viral factors and modifications were suggested to promote more compact helical assembly with additional interactions for virion packaging (Fig. 6).^21,25^

NSV nucleocapsids have been previously demonstrated to transit between different forms during infection, replication and packaging processes (Fig. 6).^42,43^ During the preparation of this manuscript, Gonnin and coworkers have reported several cryo-EM structures of different RSV N-RNA complexes, among which a major finding of the non-canonical RSV helical nucleocapsid-like assembly of ~1.5-turn with 16 N protomers is consistent with our cryo-EM structure of one helical turn of 10 N protomers.^44^ Previous study demonstrated that the helical NC is the most biological and functional relevant form in RSV, whereas ring-like N-RNA complexes have also been observed in tomography.^42^ We did not observe any ring-like or double ring-like N-RNA complexes possibly because we used human cell expression system, which may result in variations in protein folding, post-translational modifications and overall protein assembly compared to insect cell expression system used in the other study. Nonetheless, both structural and molecular details of RSV nucleocapsid-like assembly provide fundamental information for additional functional experiments in order to further validate the critical role of nucleocapsid assembly in RSV viral transcription and replication, which could serve as a potential target for antiviral therapeutic development.

## Materials and methods

### Plasmid construction, cell culture and protein expression

The RSV N protein (GenBank accession AY911262.1) was cloned into the pCAG-OSF vector with two N-terminal strep-tag II between *xho* I and *kpn* I by ClonExpress^®^ II one step cloning kit (Vazyme). This plasmid was transfected into HEK293F cells (2 × 10^6^) with polyethylenimine linear (PEI, MW25000, Polysciences, cat: 23966-1). Protein expression was performed in 1 L erlenmeyer flasks with 300 mL of expression medium (Gibco, FreeStyle^TM^ 293 expression medium, cat: 2446428) at 37 °C, 5% CO_2_ and 130 rpm for 3 days. The cells were harvested by centrifugation at 3000 × g for 10 min at 4 °C and subsequently washed with phosphate buffered saline (PBS). Following a second centrifugation step, the cells were frozen and stored at −80 °C until purification.

### Protein purification

Cells were harvested and resuspended in lysis buffer containing 50 mM Tris-HCl pH 7.4, 150 mM NaCl, 1 mM phenylmethylsulfonyl fluoride (PMSF) and 10 mM EDTA. Cells were lysed by dry ice-methanol freeze-thaw of three times and the lysate was centrifugated at 18,000 × g at 4 °C for 60 min at 4 °C to remove the precipitate. After centrifugation, supernatant was filtered with a 0.22 μm filter and then was loaded onto StrepTrap^TM^ HP column (Cytiva) which pre-equilibrated with binding buffer containing 50 mM Tris-HCl pH 7.4, 150 mM NaCl and 10 mM EDTA. The supernatant was incubated with Strep Sepharose (Cytiva) for about 1 h, and the bound protein was eluted with buffer containing 50 mM Tris-HCl (pH 7.4), 150 mM NaCl, 10 mM D-desthiobiotin and 10 mM EDTA. The protein of interest was concentrated to approximately 1 mL and further purified by size-exclusion chromatography (Superdex 200 Increase 10/300, Cytiva) in a storage buffer containing 50 mM Tris-HCl (pH 7.4), 150 mM NaCl and 10 mM EDTA. The peak fractions containing the protein were determined by Coomassie staining SDS-PAGE (15%, w/v).

### Negative staining EM

A total of 3 μL of RSV-N protein sample at a concentration of 1 mg/mL was applied onto the glow-discharged (40 s) 300-mesh copper grid coated with a continuous carbon film (Quantifoil Micro Tools GmbH), and then incubated for 60 s to ensure the adsorption of the sample onto the copper grid. The grid was blotted from sideways with a piece of filter paper and washed with 5 µL of storage buffer before staining with 0.75% uranyl formate (UF) for 40 s. The grid was dried naturally after using filter paper to remove the excess solution, and it was then stored until imaging. The grid was positioned on a side-entry holder and loaded into a JEM-1400 operated at 120 kV, condenser lens aperture 150 μm and spot size 1. The images were taken using RADIUS software on a Morada G3 CCD camera at a magnification of 120,000 × (corresponding to a calibrated sampling of 3.23 Å per physical pixel).

### Cryo-EM sample preparation and data acquisition

Cryo-EM samples were prepared at 4 °C and in 100% humidity. A total of 3 μL of the RSV-N protein sample was applied on to glow-discharged (45 s) Quantifoil Cu R1.2/1.3 (200 mesh) grids (Quantifoil Micro Tools GmbH). The grids were blotted with filter paper for 2.5 s without blot drift, and then instantly frozen in liquid ethane using a Vitrobot Mark IV (Thermo Fisher). The frozen grids were loaded into a Titan Krios cryo-EM (Thermo Fisher) operated at 300 kV, condenser lens aperture 50 μm and spot size 5. The magnification of the microscope was set at 165,000 × (corresponding to a calibrated sampling of 0.85 Å per physical pixel). Movie stacks were collected automatically using EPU software (version 2.9.0.1519REL) on K2 direct electron camera equipped with 20 eV Gatan BioQuantum energy filter for imaging. The data acquisition operated in counting mode at a recording rate of 5 raw frames per second and a total exposure time of 6 s, and resulted 30 frames per stack and a total dose of 62.9 e^-^/Å^2^. In total, 9256 frames were obtained with defocus values spanning between −0.6 and −2.8 μm.

### Cryo-EM data processing

These raw frames were motion corrected by MotionCor2.^45^ Following the CTF correction using CTFFIND4,^46^ 9244 micrographs were submitted to EMAN2^47^ and Relion3.1^48^ to evaluate and manually pick the helical tubes. A total of 158,818 segments (528 pixels × 528 pixels with 5 asymmetric units (ASUs) between segments, which equals to 1,003,455 ASUs) were extracted in Relion3.1. Following 2D classifications in cryoSPARC3.1,^49^ the high-quality classes determined by visual inspection were subjected to two rounds of ab-initio reconstruction and heterogeneous refinement to eliminate poor-quality particles. Multiple rounds refinements were performed for optimal class including homogeneous refinement, non-uniform refinement and local refinement, and a local focus refinement is performed on regions with poor density to improve the overall resolution. The ultimate sharpened map with a 3.96 Å global resolution was estimated using the 0.143 criterion of the Fourier shell correlation (FSC) curve. The ultimate 3D map was visualized in UCSF Chimera^50^ and ChimeraX.^51^

### Segmentation, flexible fitting and modeling

An approximate segmentation of each RSV-N protein monomer was obtained by segmenting the reconstructed density of the helical tubes using the Segger plugin in UCSF Chimera. The crystal structures of RSV-N (PDB ID: 2wj8) were then rigidly fitted by alignment to segments corresponding to individual RSV-N in UCSF Chimera. Then, the model was adjusted manually in Coot^52^ as needed. The models were refined using Phenix real-space-refine, resulting a model - map correlation coefficient (CCmask) of 0.82.^53^ The final model was validated by MolProbity.^54^ The interactions were analyzed by the PDBsum web server (http: //www.ebi.ac.uk/thornton-srv/databases/pdbsum/Generate.html)^55^ and Chimera.^50^

### Supplementary information

Cryo-EM structure of the nucleocapsid-like assembly of respiratory syncytial virus

## Data Availability

The final cryo-EM maps and related atomic coordinate models of the complete RSV N-RNA complex have been deposited in the wwPDB OneDep System under EMD accession code 35728 and PDB ID code 8IUO.
