# Supplementary material for: Cryo-EM structure of the nucleocapsid-like assembly of respiratory syncytial virus
Source: Signal Transduct Target Ther. 2023 Aug 22;8:323. doi: 10.1038/s41392-023-01602-5 (PMC10444854; doi:10.1038/s41392-023-01602-5)
Supplement: Supplementary file 1 — Cryo-EM structure of the nucleocapsid-like assembly of respiratory syncytial virus [file 41392_2023_1602_MOESM1_ESM.docx]

Supplementary Materials for

**Cryo-EM structure of the nucleocapsid-like assembly of respiratory syncytial virus**

Yan Wang^1,§^, Chong Zhang^1,§^, Yongbo Luo^1^, Xiaobin Ling^1^, Bingnan Luo ^1^, Guowen Jia ^1^, Dan Su^1^, Haohao Dong^1^, Zhaoming Su^1,^*

^1^The State Key Laboratory of Biotherapy, Frontiers Medical Center of Tianfu Jincheng Laboratory, Department of Geriatrics and National Clinical Research Center for Geriatrics, West China Hospital, Sichuan University, Chengdu, Sichuan 610044, China.

§These authors contributed equally

*****Correspondence should be addressed to Z.S. (zsu@scu.edu.cn).

**This PDF file includes:**

Supplementary table. S1

**Supplementary Table 1.** Cryo-EM data collection, processing, and model refinement statistics in RSV-N-RNA complex.

| **Cryo-EM data collection and processing** | |
| --- | --- |
| Voltage (kV) | 300 |
| Microscope | Titan Krios |
| Voltage (kV) | 300 |
| GIF Quantum energy filter slit width (eV) | 20 |
| Detector | Gatan K2 |
| Nominal Magnification | 165,000 × |
| Pixel size (Å) | 0.85 |
| Symmetry imposed | C1 |
| Defocus range (μm) | -0.6 – -2.8 |
| Electron exposure (e^-^/Å^2^) | 62.9 |
| Micrographs (acquired/used) | 9256/9244 |
| Number of extracted particles | 158,818 |
| Number of particles after 2D classifications | 89,405 |
| Number of particles going to 3D refinement | 42,956 |
| Map resolution at 0.143 FSC criterion (Å) | 3.96 |
| Local resolution range (Å) | 3.689 - 9.551 |
| Sharpening B-factor (Å^2^) | -85.3 |
| **Model refinement** | |
| Atoms | 14785 |
| Residues | Protein:1810 Nucleotide:36 |
| CC_mask_ | 0.82 |
| Resolution_FSC map vs. model @ 0.5_ (Å) | 4.1 |
| r.m.s. deviations | |
| Bond lengths (Å) | 0.003 |
| Bond angles (°) | 0.734 |
| Clash score | 3 |
